# Effect of *Nardostachys jatamansi* DC. on Apoptosis, Inflammation and Oxidative Stress Induced by Doxorubicin in Wistar Rats

**DOI:** 10.3390/plants9111579

**Published:** 2020-11-15

**Authors:** Mhaveer Singh, Mohammad Ahmed Khan, Kamal Y. T., Javed Ahmad, Usama A. Fahmy, Sabna Kotta, Nabil A. Alhakamy, Sayeed Ahmad

**Affiliations:** 1 School of Medical and Allied Sciences, G D Goenka University, Haryana 122103, India; mhaveer.singh@gdgoenka.ac.in; 2 Department of Pharmacology, School of Pharmaceutical Education & Research, Hamdard University, Hamdard Nagar, New Delhi 110062, India; drm.ahmedkhan@jamiahamdard.ac.in; 3 Department of Pharmacognosy, College of Pharmacy, King Khalid University, Abha 611441, Saudi Arabia; kamal.yt@gmail.com; 4 Department of Pharmaceutics, College of Pharmacy, Najran University, Najran 11001, Saudi Arabia; jaahmed@nu.edu.sa; 5 Department of Pharmaceutics, Faculty of Pharmacy, King Abdulaziz University, Jeddah 21589, Saudi Arabia; uahmedkauedu.sa@kau.edu.sa (U.A.F.); skotta@kau.edu.sa (S.K.); nalhakamy@kau.edu.sa (N.A.A.); 6 Center of Excellence for Drug Research & Pharmaceutical Industries, King Abdulaziz University, Jeddah 21589, Saudi Arabia; 7 Bioactive Natural Product Laboratory, School of Pharmaceutical Education & Research, Hamdard University, Hamdard Nagar, New Delhi 110062, India

**Keywords:** biochemical estimation, cardioprotection, cardiotoxicity, doxorubicin, lipid peroxidation, *Nardostachys jatamansi* DC.

## Abstract

The study aimed to investigate the protective action of jatamansi (*Nardostachys jatamansi* DC.) against doxorubicin cardiotoxicity. Methanolic extract of jatamansi (MEJ) was prepared and standardized using HPTLC fingerprinting, GC-MS chemoprofiling, total phenolic content, and antioxidant activity in vitro. Further in vivo activity was evaluated using rodent model. Animals were divided into five groups (*n* = 6) namely control (CNT) (Normal saline), toxicant (TOX, without any treatment), MEJ at low dose (JAT1), MEJ at high dose (JAT2), and standard desferrioxamine (STD). All groups except control received doxorubicin 2.5 mg per Kg intra-peritoneally for 3 weeks in twice a week regimen. After 3 weeks, the blood samples and cardiac tissues were collected from all groups for biochemical and histopathological evaluation. Treatment with MEJ at both dose levels exhibited significant reduction (*p* < 0.001 vs. toxicant) of serum CK-MB (heart creatine kinase), LDH (Lactate dehydrogenase) & HMG-CoA (3-hydroxy-3-methylglutaryl-coenzyme A) levels, and tissue MDA (melondialdehyde) level; insignificant difference was observed (*p* > 0.05) in TNF-alpha (tumour necrosis factor), IL-6 (interleukine-6) levels and caspase activity as compared to TOX. Histopathological evaluation of cardiac tissues of different treatment groups further reinforced the findings of biochemical estimation. This study concludes that jatamansi can protect cardiac tissues from oxidative stress-induced cell injury and lipid peroxidation as well as against inflammatory and apoptotic effects on cardiac tissues.

## 1. Introduction

Doxorubicin (DOX) is a clinically useful anticancer drug being used in the management of different types of cancers including lymphomas and carcinomas [1,2]. However, the major problem associated with its clinical use is the development of dose-dependent cardiotoxicity [2]. DOX cardiotoxicity can occur acutely (within 3 days) upon administration whereas chronic toxicity is seen within 30 days of the last dose. The exact cause of DOX-induced preferential cardiotoxicity is poorly understood despite exhaustive research and investigation [3]. Different lines of evidence, however, obtained through preclinical studies have proposed a number of mechanisms for cardiotoxicity of DOX including the induction of oxidative stress [4], cardiomyocyte apoptosis at acute as well as chronic levels [5,6,7,8], iron mediated stress [9], adrenergic dysfunction, and abnormal handling of calcium [10,11,12]. However, DOX-induced cardiotoxicity has been shown to be induced through pathways independent of cytotoxic mechanisms. Therefore, a strategy can be developed for protecting against doxorubicin-induced cardiotoxicity without affecting the therapeutic value of the drug [13].

**Figure 1:**
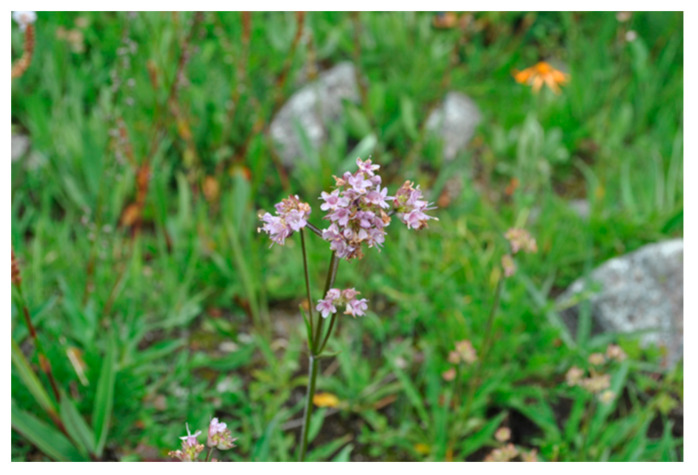
*Nardostachys jatamansi*.

*Nardostachys jatamansi* DC., commonly known as jatamansi, is an important medicinal herb of Indian origin, belonging to the family Caprifoliaceae. Jatamansi belongs to valerian subfamily, which includes herbaceous flowering plants, which sometimes have a characteristic odor (Figure 1). It is an important part of various traditional systems of medicines to treat different disorders [14,15,16,17]. It has been used in traditional medicines for the treatment of cardiac and many other diseases. It is reported to possess anti-hypertensive, antispasmodic, sedative, and anxiolytic activity. Its use for the treatment of hyperglycemic and inflammatory conditions has also been reported in traditional literature [18]. Various sesquiterpenes such as lignans and neolignans are present in root extracts of plants [19] and have been suggested to protect cells and tissues through their antioxidant properties [20]. Recently, in a rodent model of inflammation, jatamansi has been shown to inhibit the production of interleukin-1 (IL-1), interleukin-6 (IL-6), tumor necrosis factor (TNF-alpha), and interferon (IFN-α/β) by inhibiting MAPKs (Mitogen-activated protein kinase) activation and induction of IRF (interferon regulatory factor) [21]. However, the drug has reported protection against doxorubicin-induced lipid peroxidation [22,23]; this study is, however, designed to evaluate the possible protective role of standardized jatamansi extract on oxidative, inflammatory and apoptotic markers against DOX-induced cardiotoxicity in rats.

## 2. Materials and Methods

### 2.1. Chemicals and Reagents

Catechin (98%), ascorbic acid (99%) and 2,2-diphenyl-1-picrylhydrazyl (DPPH) were purchased from Sigma Aldrich, Missouri, USA. Sodium nitroprusside, naphthylethylene diamine dihydro chloride and sulphanilamide were provided by SRL Mumbai, India. Folin-Ciocalteau (FC) reagent, sodium carbonate (Na_2_CO_3_), 5,5′-dithio-bis (2-nitrobenzoic acid) (DTNB), thiobarbituric acid (TBA), and trichloro acetic acid (TCA) were purchased from S. D. Fine, India. DOX and desferrioxamine were obtained as commercial formulations from local Pharmacy, New Delhi.

### 2.2. Plant Material

The rhizomes of jatamansi were purchased from local herbal drug market (Kharibawli, Old Delhi), which was identified and authenticated by Dr. HB Singh [Scientist F & Head Raw Materials Herbarium & Museum, NISCAIR (National Institute of Science Communication and Information Resources), Dr. K. S. Krishnan Marg (Near Pusa Gate), New Delhi 110 012, Voucher specimen No. NISCAIR/RHMD/Consult/-2008-09/1149/181/02/01-08].

### 2.3. Extract Preparation

The aqueous methanolic extraction of Jatamansi (MEJ) was done using dried and coarsely powdered rhizome (200 gm). The hot extraction was carried out using 70:30 *v/v* aqueous methanol with a reflux condenser in a water bath for 2 h. The process was repeated three times to obtain complete extraction by adding fresh solvent and collecting the extracts. The extracts were pooled and concentrated using rota-vapour and further dried by lyophilisation (extractive value 8.76 % *w/w*).

### 2.4. Standardization of MEJ

#### 2.4.1. HPTLC Profiling

The MEJ was reconstituted in methanol (20 mg mL^−1^), filtered through 0.45 μm membrane filter, and HPTLC profiling was done on silica using toluene: ethyl acetate (73: 7, *v/v*) as a solvent system. The plate was air dried and scanned at 254 nm wavelength.

#### 2.4.2. GC-MS Chemoprofiling

The lyophlised MEJ powder was dissolved in GC-MS grade hexane (1.0 mg mL^−1^) and filtered. The hexane soluble constituents were GC-MS analysis on Agilent 7890A GC system coupled with 5975C inert XL E1/C1 MSD model # G3174A, Agilent Technologies, Wilmington, DE, USA mass spectroscopic system having 30 m (length) × 250 μm × 0.25 μm HP-5MS capillary column and autosampling system. The injector was operated in splitless mode with 1.0 µL injection volume. The gas flow rate was maintained at 1.0 mL min^−1^, while inlet temperature was maintained at 270 °C. Oven temperature was initially kept at 40°C for 1 min and then increased to 140 °C at a heating rate of 4.0 °C min^−1^followed by 200 °C at a heating rate of 10 °C min^−1^ and lastly up to 280 °C at a heating rate of 8 °C min^−1^. The total run time was 42 min with MS operated in SCAN mode.

#### 2.4.3. Determination of Total Phenolic Content and In Vitro Antixidant Potential

The total phenolic content in MEJ was estimated using Folin–Ciocalteau reagent, and catechin was used as a standard [24]. However, free radical scavenging potential of MEJ was determined for concentrations from 5.0 to 100 μg mL^−1^ by DPPH method [25]. The 1 mL of DPPH solution (0.004%, *w/v*) in 95% methanol was taken in test tubes, and then 1.0 mL of MEJ was added followed by serial dilutions (5.0–100 μg mL^−1^) to every test tube. Ascorbic acid was used as a reference standard and dissolved in methanol to make the stock solution with the same concentration (1.0 mg mL^−1^) followed by serial dilutions (5.0–100 μg mL^−1^). The absorbance was measured after 10 min at 515 nm. The control sample was prepared containing the same volume without any extract and reference ascorbic acid, whereas methanol (95%) was used as blank. The percentage scavenging activity of the MEJ against DPPH free radical was measured using the following Equation (1):

% Inhibition = [(A0−A1)/A0] × 100(1)

where A0 was the absorbance of the control (blank, without extract), and A1 was the absorbance of the extract or standard.

Similarly, Nitric oxide (NO) radical scavenging activity of MEJ was determined as per the method reported by Liu et al. (2009) [26]. The method is based on the principle that sodium nitroprusside in aqueous solution at physiological pH spontaneously generates nitric oxide, which interacts with oxygen to produce nitrite ions; it can be determined by the use of the Griess Illosvoy reaction where scavengers of nitric oxide compete with oxygen, leading to reduced production of nitric oxide.

### 2.5. In Vivo Study

#### 2.5.1. Animals

Adult male Wistar rats 10–12 weeks old weighing 200–250g were used in the study, which was approved by Institutional Animal Ethics Committee in the CPCSEA (Committee for the purpose of control and supervision of experiment on animals) proposal No. 659 obtained from Central Animal House facility of Jamia Hamdard (Hamdard University). All animals were maintained under standard laboratory conditions with standard pellet diet and free access to water. The animal rooms were maintained at 20–25 °C with a 12 h light/dark cycle. All animals were subjected to humane treatment, and the study was conducted in accordance with the strict guidelines of Institutional Ethics Committee. Animals were divided into five groups (*n* = 6) namely control (CNT, group 1), toxicant (TOX, group 2), MEJ low dose (JAT1, group 3), MEJ high dose (JAT2, group 4), and standard (STD, group 5). The MEJ was given in two low and high doses (250 and 500 mg mL^−1^ Kg^−1^ body weight day^−1^) as both the doses were found to bw effective against parkinson and stress in rats [27,28].

#### 2.5.2. Treatment Schedule

The CNT group was given normal saline orally at 1.0 mL/kg/day for 3 weeks. TOX group was administered with DOX 2.5 mg Kg^−1^ body weight through intra-peritoneal route twice a week for 3 weeks along with normal saline 1.0 mL/kg/day [29,30]. Test groups were treated with JAT1 (250 mg/kg/day) and JAT2 (500 mg/kg/day) for 3 weeks along with DOX as given for toxic control. The desferrioxamine (Desferal injection) was used as standard (STD) and administered (i.v.) 50 mg/kg/day for 3 weeks along with DOX. The animals were anaesthetised 72 h after the last dose of DOX, and blood was withdrawn from the retro-orbital plexus. The blood was allowed to clot and serum was separated and stored for biochemical estimation. Further, animals were killed under high dose of anaesthesia; hearts were excised and immediately washed with ice cold saline for tissue estimations. For histopathological evaluation, the heart tissues of different groups were fixed in 10% neutral buffered formalin. The serums obtained were used for analysis of different biochemical markers, whereas heart tissue homogenate were utilised for the analysis of total protein and marker enzymes. The histopathological changes were observed using heart tissues of all groups at different magnifications after proper staining.

#### 2.5.3. Biochemical Estimation

CK-MB and LDH activity was measured in serum using available kits (Reckon Diagnostics Pvt. Ltd., Vadodara, India) as per the reported method [31]. HMG-CoA was estimated using ELISA kit (Cusa Biotech Pvt. Ltd., New Delhi, India). TNF-alpha was estimated in cardiac tissue using commercial ELISA kit (e-Bioscience, Inc., San Diego, CA, USA) as per the method described by Lehmann et al. (2008) [32]. Quantitative measurement of IL-6 was performed in vitro in serum using Elisa kit (Ray Biotech, Inc., USA) as per the method described by Helle et al. (1991) [33]. Caspase-3 activity was measured in heart tissue using Capase-3/CPP32 colorimetric ELISA assay kit (Bio Vision, Inc., California, USA) as discussed by Jaeschke et al. (1998) [34]. Similarly, estimation of lipid peroxidation was done by the method reported by Iqbal et al. (2008) [35] and total protein estimation in both serum and heart tissue as per the method followed by Lowry et al., 1951 [36].

### 2.6. Histopathology

Heart tissue sections were stained by Haematoxylin and Eosin stain, and histopathological evaluation was carried out by a pathologist blinded to the treatment [37].

### 2.7. Statistical Analysis

Graph pad Prism3.0 (Graph pad; San Diego, CA, USA) was used for statistical analysis of results. All results were expressed as mean ± standard deviation (SD). Data from different groups were compared with the analysis of variance (ANOVA) followed by Tukey test. Values were considered statistically significant when *p* < 0.05.

## 3. Results

### 3.1. Standardization of MEJ

**Figure 2:**
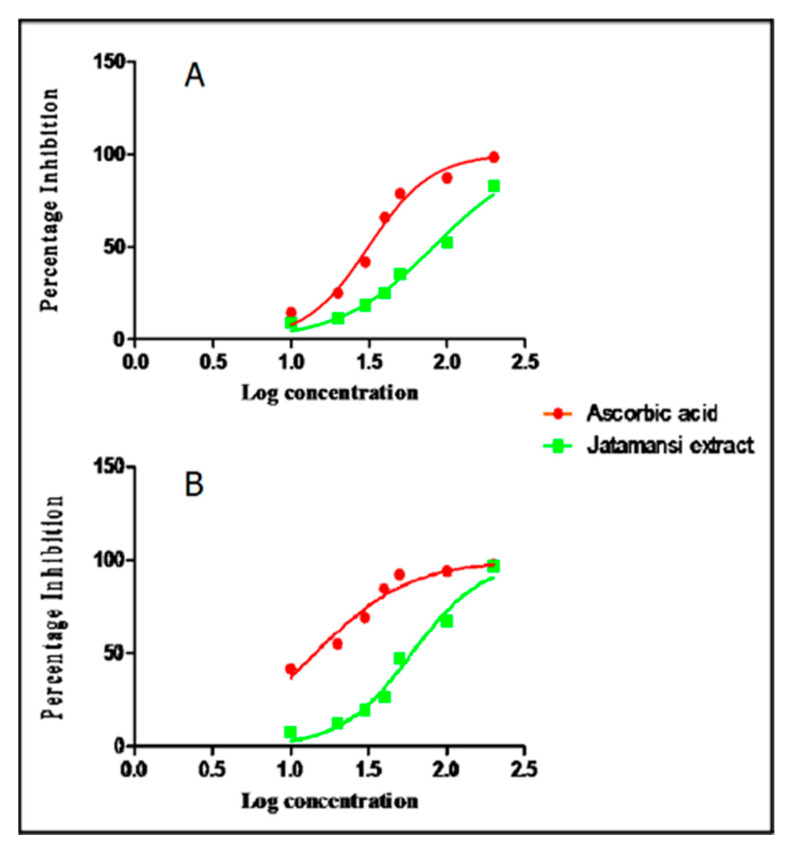
Antioxidant potential of MEJ compared with ascorbic acid by using DPPH (**A**) and Nitric oxide (**B**) scavenging method.

**Figure A1:**
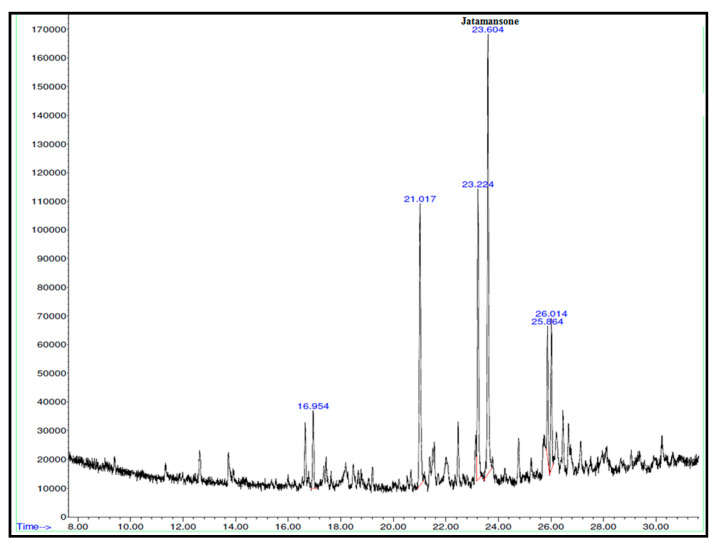
GC-MS chromatogram of hexane fraction of MEJ showing jatamansone as main constituent.

**Figure A2:**
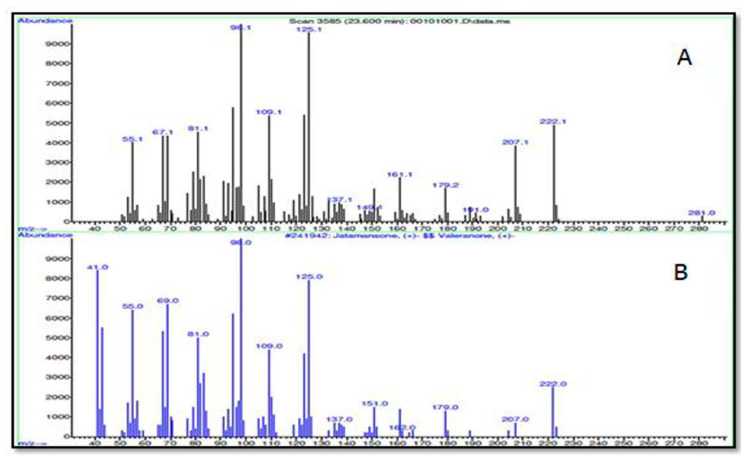
Mass spectra of jatamansone in sample (**A**) comparing with library (**B**) by GC-MS.

The HPTLC profiling of MEJ at 254 nm (Figure S1 in Supplementary Material) showed the presence of 10 well-separated spots corresponding to 10 different compounds at different R_f_. On the other hand, GC-MS chemo profiling for the hexane soluble fraction of MEJ led to separation and identification of six major constituents. Of these six constituents, jatamansone was present in the highest concentration (32.05%) (Table 1, Figure A1 and Figure A2 in Appendix A). The phenolic compounds reported an inhibitory effect on carcinogenesis and mutagenesis in humans [38]; the total phenolic content in the MEJ was found to be 4.12 ± 0.09%, *w/w*. The MEJ showed antioxidant action by inhibiting DPPH radical in a concentration-dependent manner with an IC_50_ values of 81.99 μg mL^−1^. Ascorbic acid, which was used as the standard, exhibited an IC_50_ value of 31.36 μg mL^−1^ (Figure 2A). Similarly, MEJ also showed concentration-dependent nitric oxide scavenging activity at the concentration of 10–200 μg mL^−1^ with an IC_50_ value of 60.03 μg mL^−1^ (Figure 2B). Ascorbic acid was also found to possess nitric oxide scavenging activity with an IC_50_ value of 14.44 μg mL^−1^. RESULTS OF GC-MS PROFILING OF MEJ.

### 3.2. In Vivo Study

#### Biochemical Estimation

**Figure 3:**
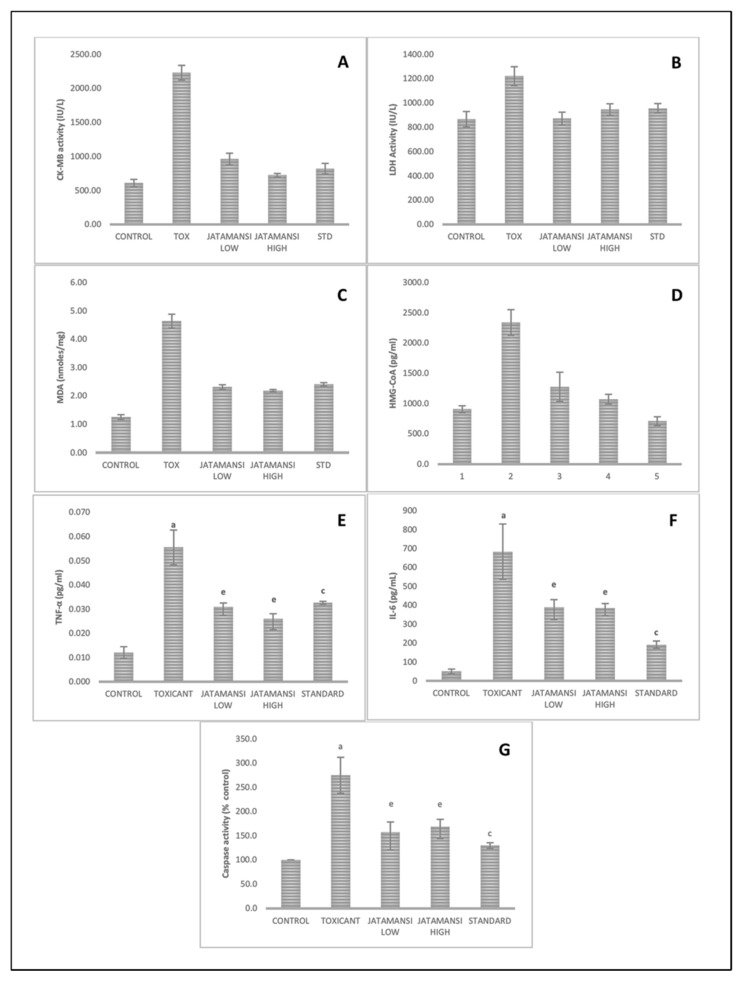
Results of different biochemical parameters for CNT group, TOX group, STD, MEJ 250 mg/kg (JAT1), and 500 mg/kg treated group (JAT2). (**A**) serum CK-MB level where TOX group showed a significant increase (*p* < 0.01 vs. Control) in CK-MB activity, indicating myocardial damage. Treatment with Jatamansi exhibited a significant reduction in CK-MB activity. (**B**) serum LDH activity was significantly increased with DOX treatment. This effect was reversed upon treatment with *Jatamansi.* (**C**)Tissue MDA (TBARS) level was elevated significantly compared to control group, which indicates severe lipid peroxidation and oxidative stress. Jatamansi at both dose levels prevented elevation of MDA levels significantly; (**D**) serum HMG-CoA level, which was similar to other mentioned parameters, was significantly low in Jatamansi-treated animals; Serum inflammatory marker TNF-α (**E**) and IL-6 (**F**) also showed elevated levels upon DOX treatment. The anti-inflammatory effect of Jatamansi was evident from lowering of TNF- α and IL-6 levels as compared to DOX. (**G**) % caspase activity measured as marker of apoptosis showed a significant increase in cardiac tissue of DOX-treated animals.

Results of CK-MB estimation in the serum of different experimental groups showed that CK-MB level in TOX group was significantly elevated (*p* < 0.001vs. CNT). Treatment with MEJ and STD caused significant reduction in the elevated CK-MB level (*p* < 0.001 vs. TOX) (Figure 3A). Similarly, TOX group treatment caused an elevation in the mean serum LDH level (*p* < 0.001vs. CNT), while treatment with either MEJ or STD showed a significant reduction in the elevated LDH level (*p* < 0.001 vs. TOX) (Figure 3B). Mean tissue MDA levels estimated as a measure of lipid peroxidation (nmol mg^−1^ of protein) were found to be significantly elevated in the TOX group (*p* < 0.001vs. CNT). However, a significant reduction of MDA levels was observed upon treatment with MEJ and STD (*p* < 0.001vs. TOX) (Figure 3C). TOX group showed significant elevation in HMG-CoA levels (*p* < 0.001vs. CNT). However, MEJ- and STD-treated groups caused a significant reduction in elevated HMG-CoA level (*p* < 0.001 vs. TOX) (Figure 3D). In agreement with the previous observations, TOX treatment also caused significant elevation in cardiac tissue caspase-3 activity, serum IL-6 and TNF-alpha levels (*p* < 0.001 vs. CNT). Whereas, MEJ treated groups at 250 and 500 mg kg^−1^ showed significant reduction in (*p* < 0.05) in the levels of these cytokines in comparison to the TOX group. STD group also showed a significant reduction (*p* < 0.001vs. TOX) of TNF-alpha, IL-6 as well as caspase-3 levels compared to TOX (Figure 3E–G).

### 3.3. Histopathology

**Figure 4:**
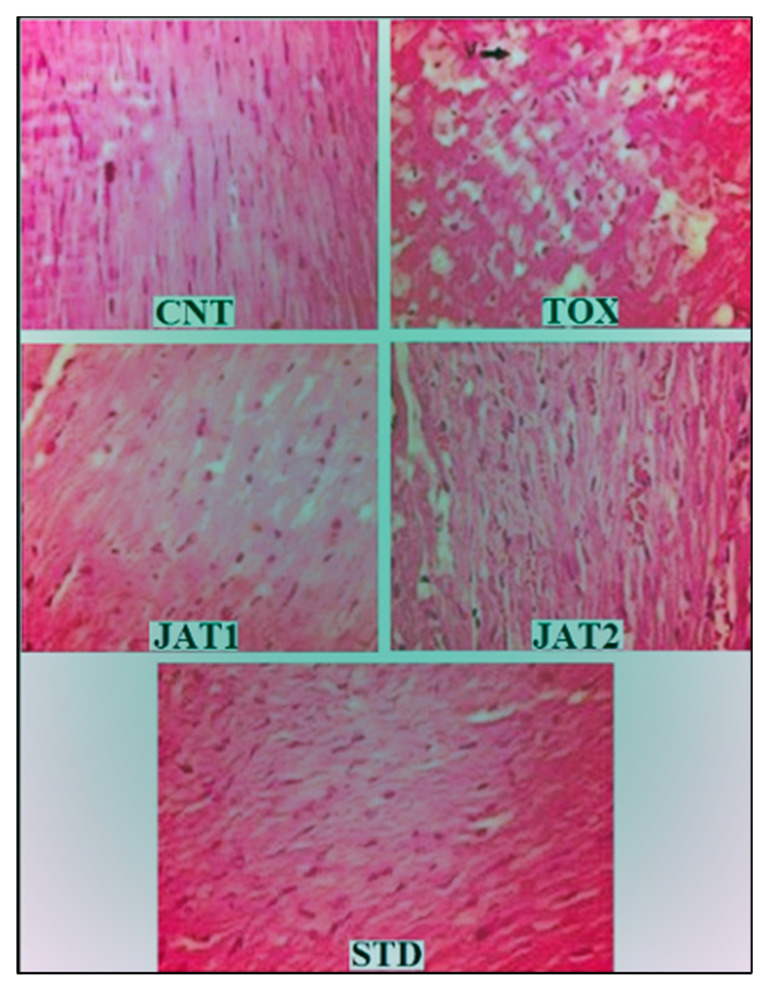
Histopathology of cardiac tissues from animals belonging to CNT, TOX group, MEJ groups, and STD-treated group was assessed with special reference to the integrity of myocardial fibre integrity and evidence of cardiac damage caused by DOX. CNT shows normal myocardial structure, whereas TOX shows disarray of myocardial cells with small and large vacuolar myopathy but no evidence of necrosis of the myocardium seen. Cardiac tissue from MEJ (JAT1, JAT 2) and STD groups, respectively, shows normal myocardial architecture.

Samples from cardiac tissues from animals belonging to CNT, DOX, MEJ (JAT1 and JAT2), and STD treatment groups were examined with special reference to histological evidence in reference to DOX-induced cardiac damage. Normal myocardial tissue architecture was observed in the CNT group. On the other hand, myocardial tissue from TOX group exhibited disarrayed fibres and vacuolar myopathy with evidence of necrosis. The large disarray and vacuolar myopathy were not observed in the myocardial tissue samples from MEJ-treated (250 and 500 mg/kg/day) as well as STD treatment groups (Figure 4).

## 4. Dicussion

In the present study, MEJ was prepared and standardized using HPTLC fingerprinting, GC-MS profiling and total phenolic content estimation. In vitro antioxidant efficacy of extract was evaluated by DPPH assay and free radical scavenging test using nitric oxide. This standardized extract was further used for in vivo study to evaluate the possible effect of jatamansi in murine model of DOX cardiotoxicity by using oxidative stress, and inflammatory and apoptotic markers. Biochemical and histopathological changes observed upon administration of 15 mg/kg of DOX in divided doses over 3 weeks provided confirmatory evidence of its cardiotoxic potential.

DOX treatment for induction of cardiotoxicity also elevated CK-MB level by more than threefold. Increased CK-MB activity indicates myocardial infarction and rhabdomylosis. The present experiment showed that there was a significant increase in LDH level of DOX intoxicated rats. An increase in LDH level indicates cardiovascular damage induced by DOX and its metabolites. Lipid peroxidation was measured as nmol of MDA mg^−1^ of protein. Significant elevation in the MDA level was observed in the TOX group. Higher MDA levels can be due to DOX-induced oxidative damage.

Interleukin-6 plays a dual role both as a pro-inflammatory as well as anti-inflammatory cytokine. DOX is reported to cause IL-6 release in addition to TNF-α and many mediators of apoptosis. These factors are believed to be responsible for its toxicity in the cardiac tissue [39]. We are reporting elevation of interleukin-6 level upon treatment with DOX as compared to the CNT group (*p* < 0.001). Wang et al. reported increased expression of interleukin 6 in the kidney tissue of DOX-treated rats [40]. These evidences suggest that the pro-inflammatory effect of DOX involves IL-6. Van der Veen et al. 2000 have reported the role of TNF-alpha in augmenting intra-tumoral concentration of DOX but the results do not define the role of TNF-alpha in modulating the DOX activity in vivo [41]. Chiosi et al. have reported that TNF-alpha activity is modulated by DOX in cardiomyocited to induced apoptosis, indicating the role of TNF-alpha signaling in DOX cardiotoxicity [42]. Results of our study revealed that DOX showed significant elevation in TNF-alpha level (*p* < 0.001) compared to the CNT group. TNF-alpha concentration increases in case of heart failure and is an independent predictor of mortality [43]. This showed that increased TNF-alpha level in the DOX-treated group could be due to induction of heart failure by DOX.

Results of estimation of apoptosis-specific enzyme caspase-3 activity in our study showed significant increase after DOX treatment. Previously, Sharma et al., confirmed that DOX-induced cardiotoxicity is mediated via caspase-3-dependent apoptotic pathway.

Treatment with test drug i.e., MEJ at the dose level of 250 and 500 mg kg^−1^ day^−1^ orally up to 21 days significantly reduced elevated CK-MB levels (*p* < 0.001) in DOX-intoxicated rats. Treatment with MEJ 250 mg kg^−1^ significantly reduced elevated levels of MDA compared to TOX group, whereas treatment with MEJ 500 mg kg^−1^ also significantly reduced elevated levels of MDA compared to the TOX group. This indicated reduced lipid peroxidation in MEJ-treated groups. IL-6 is an interleukin, which has complex action on inflammatory pathways and is secreted by T cells and macrophages in response to inflammatory conditions. Elevated IL-6 levels are observed in DOX treatment in our study. This confirmed the previous studies, which reported that DOX cardiotoxicity is also mediated by inflammatory cytokines. Treatment with MEJ 250 and 500 mg kg^−1^ for 3 weeks caused reduction in IL-6 levels. TNF-alpha is a mediator of acute phase systemic inflammation and is reported to play a role in DOX-induced inflammation. Similar to IL-6, TNF-alpha level in MEJ group at 250 and 500 mg/kg showed significant reduction compared to TOX group. Elevated TNF-alpha levels were observed even after treatment with MEJ extract at 250 and 500 mg kg^−1^.

Caspase-3 activity, measured as a pro-apoptotic marker, after treatment with MEJ at a dose of 250 mg kg^−1^, showed significant difference compared to the TOX group. This indicated that caspase-3-mediated pro-apoptotic pathways were inhibited by MEJ at 250 mg kg^−1^ in DOX-treated rats.

Previously, HMG-CoA reductase inhibitor has been reported to protect against DOX-induced cardiotoxicity. Lovastatin, one of the HMG-CoA inhibitors, has been reported to have a synergistic effect with DOX in ovarian cancer cells. HMGCoA has also been implicated in the generation of ketone bodies. HMG-CoA is converted to acetoacetate, and acetyl-CoA by HMG-CoA lyase. The acetoacetate so formed is β-hydroxybutyrate, which is the ketone body present in highest amount in the body. While under normal physiological conditions, such ketone bodies perform multiple roles including energy provision. Excess ketone bodies formation is associated with free radical generation, oxidative stress and may lead to lipid peroxidation [44]. Since DOX is also reported to upregulate cholesterol transporter level in cardiac cholesterol transporter level [45], the association between DOX-induced oxidative stress and elevated HMGCoA levels warrant further study. Our study is the first to report the effect of DOX on serum HMG-CoA level in rats. Animals treated with MEJ were observed to have significantly lower serum HMG-CoA level than TOX group.

Further, studies are needed to investigate the mechanism and role of HMG-CoA in mediating various effects of DOX. Results of histopathology showed a protective effect of MEJ on disarrayed myocardial fibre in DOX-treated animals. This also depicted a primarily antioxidant property of the tested extract.

*Nardostachys jatamansi* contains jatamansone, jatamansic acid, lignans, and many other sesquiterpens. GC-MS analysis of MEJ showed that 32.05% jatamansone is present in the prepared extract compare to other major constituents. High jatamansone content could be implicated for the antioxidant effect of MEJ, however, studies on isolated jatamanson are needed for DOX-induced myocardial injury. Inhibition of lipid peroxidation has also been used as a measure of activity of a herbal agent like Jatamansi for a very long time [46]. Subhashini et al. reported the cardioprotective effect of jatamansi, however, the study was majorly limited to an effect on mitochondrial damage due to DOX. Subhashini et al. has also proposed an antioxidant role of jatamansi in preventing DOX mitochondrial damage. The present study provides evidence on the antioxidant and cardioprotective role of jatamansi in DOX-induced oxidative stress [22]. Results of the present study also showed that MEJ did reduce proapoptotic and inflammatory markers. So, it can be concluded from the present study that MEJ can protect cardiac tissue from oxidative stress-induced cell injury and lipid peroxidation. It also interfered with DOX-induced inflammatory and apoptotic cascades in cardiac tissue. Therefore, the present experiment justifies the further development of jatamansi as a protective agent against DOX-induced cardiotoxicity.

## Figures and Tables

**Table 1:** Results of GC-MS profiling of MeJ.

S. No	Name of Constituent	R_t_	Area Percentage	Matching Percentage
1	Seychellene	16.957	5.67	96
2	Acenaphthylene	21.019	22.36	55
3	Patchouli alcohol	23.222	21.73	98
4	**Jatamansone**	**23.606**	**32.05**	**99**
5	1-methyl-4-chloro-3,5-dimethoxy-1H-pyrazole	25.866	8.30	60
6	Illudol $$ 3,6,6,7b-tetramethyl decahydro-1H-cyclobuta[e]endene-3-ol	26.015	9.89	44

## Supplementary Materials

The following are available online at https://www.mdpi.com/2223-7747/9/11/1579/s1, Figure S1: HPTLC chromatogram (A) and developed HPTLC plate (B) of MEJ showing corresponding spot and peaks at 254 nm.

## Abbreviations

ANOVA	Analysis of Variance
CK-MB	Creatine kinase myocardial band
CPCSEA	Committee for the purpose of control and supervision of experiment on animals
DOX	Doxorubicin
DPPH	2,2-diphenyl-1-picrylhydrazyl.
ELISA	Enzyme Linke Immunosorbent Assay
GC-MS	Gas chromatography–mass spectrometry
HMG-CoA	3-hydroxy-3-methyl-glutaryl-coenzyme A
HPTLC	High-performance thin-layer chromatography
IL-6	Interleukin-6
MDA	Malondialdehyde
NO	Nitric Oxide
TBA	Thiobarbituric Acid
TNF-α	Tumor Necrosis Factor-Alpha
