# Supplementary material for: Effect of Nardostachys jatamansi DC. on Apoptosis, Inflammation and Oxidative Stress Induced by Doxorubicin in Wistar Rats
Source: Plants (Basel). 2020 Nov 15;9(11):1579. doi: 10.3390/plants9111579 (PMC7734586; doi:10.3390/plants9111579)
Supplement: Supplementary file 1 [file plants-09-01579-s001.pdf]

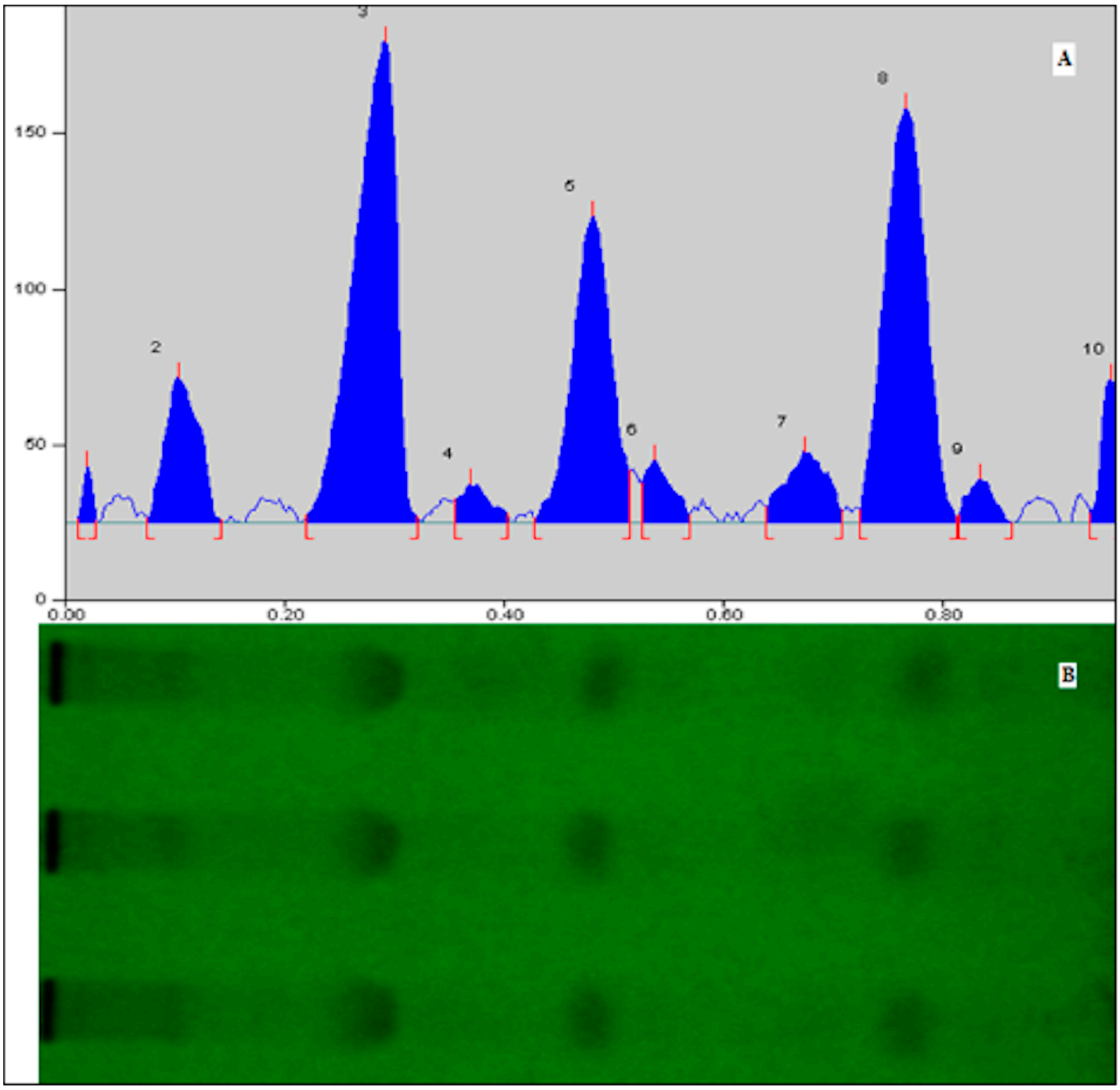

Figure S1. HPTLC chromatogram (**A**) and developed HPTLC plate (**B**) of MEJ showing corresponding spot and peaks at 254 nm.
